# Chloroform in Gonorrhea

**Published:** 1851-09

**Authors:** 


*Chloroform in Gonorrhea.*—M. Venot recommends injections of chloroform as an *abortive* treatment in gonorrhea, that is to say, of arresting it in the earliest period of its development; and thinks that it will replace the nitrate of silver, which is so frequently employed for this purpose. Pure chloroform is injected by a glass syringe, the perineum being pressed upon; the first effect is burning heat, then a sensation of cold follows. The injection does less good after the first two or three days of the gonorrhea have passed; but, employed before that time, it almost invariably arrests it. Injected into the vagina, the results were less satisfactory.—*L'Union Med.—Am. Jour. Med. Sci.*

